# A Comparative Review of Vagal Nerve Stimulation Versus Baroreceptor Activation Therapy in Cardiac Diseases

**DOI:** 10.7759/cureus.40889

**Published:** 2023-06-24

**Authors:** Akshat V Arya, Himanshi Bisht, Apoorva Tripathi, Manali Agrawal, Ashwati Konat, Jay Patel, Kamalika Mozumder, Dhrumil Shah, Devansh Chaturvedi, Kamal Sharma

**Affiliations:** 1 Internal Medicine, Byramjee Jeejeebhoy Medical College, Ahmedabad, IND; 2 Medicine, Byramjee Jeejeebhoy Medical College, Ahmedabad, IND; 3 Zoology, Biomedical Technology and Human Genetics, Gujarat University, Ahmedabad, IND; 4 Internal Medicine, Gujarat Medical Education and Research Society Medical College, Gandhinagar, IND; 5 Internal Medicine, King George Medical University, Lucknow, IND; 6 Cardiology, Kamal Sharma Cardiology Clinic, Ahmedabad, IND

**Keywords:** autonomic regulation, device therapy, cardio-neuromodulation, sympatho-vagal imbalance, vagal nerve stimulation, baroreceptor activation therapy

## Abstract

Sympathetic imbalance coupled with impairment of baroreceptor control is a key factor responsible for hemodynamic abnormalities in congestive heart failure. Vagal nerve stimulation (VNS) and baroreceptor activation therapy (BAT) are two novel interventions for the same. In this paper, we review the role of sympathovagal alterations in cardiac diseases like heart failure, arrhythmia, hypertension (HTN), etc. Studies like neural cardiac therapy for heart failure (NECTAR-HF), autonomic regulation therapy to enhance myocardial function and reduce progression of heart failure (ANTHEM-HF), and baroreflex activation therapy for heart failure (BEAT-HF), which comprise the history, efficacy, limitations, and current protocols, were extensively analyzed in contrast to one another. Vagal nerve stimulation reverses the reflex inhibition of cardiac vagal efferent activity, which is caused as a result of sympathetic overdrive during the course for heart failure. It has shown encouraging results in certain pre-clinical studies; however, there is also a possibility of serious cardiovascular adverse events if given in higher than the recommended dosage. Attenuated baroreflex sensitivity is attributed to cardiac arrhythmogenesis during heart failure. Baroreceptor activation therapy reverses this phenomenon. However, the surgical procedure for baroreceptor stimulation can have unwarranted complications, including worsening heart failure and hypertension. Considering the effectiveness of the given modalities and taking into account the inconclusive evidence of their adverse events, more clinical trials are needed for establishing the future prospects of these interventional approaches.

## Introduction and background

Heart failure is known to have an elaborate pathophysiological mechanism of manifestation. Many etiological factors are associated with the same. However, regardless of etiology, chronic autonomic imbalance with sympathetic overactivation combined with different degrees of vagal withdrawal in heart failure (HF) with reduced ejection fraction (HFrEF) is not an uncommon occurrence [1,2]. Autonomic nervous system (ANS) modulation (neuromodulation), therefore, has gained increasing popularity as a therapeutic modality [3]. This review article will focus on two such modalities: baroreflex activation therapy (BAT) and vagal nerve stimulation (VNS).

Baroreflex activation therapy (BAT), delivered by implanting a device resembling pacemaker, stimulates baroreceptors resulting in an increased parasympathetic activity, which as a reflex causes a decrease in sympathetic activity, thus aiming to restore the autonomic imbalance [4,5].

Though studies have shown BAT to be a promising novel device for HFrEF patients, it is still considered among "devices under evaluation" because not enough evidence is available for the same [5]. For more than three decades, vagal nerve stimulation (VNS) has been used clinically to modulate afferent and efferent pathways for managing patients with depression and epilepsy stating a very good safety profile [6,7]. Combined afferent and efferent stimulation have yielded functional biological results in HF patients; afferent stimulation modulates sympathetic and parasympathetic activity centrally, resulting in peripheral vasodilation, while efferent stimulation leads to anti-adrenergic effects in the cardiac nervous system and through presynaptic and postsynaptic interactions [7].

Globally, heart failure continues to have a significant prevalence, and there have been advancements in medical interventions resulting in improved survival. Nevertheless, the prevalence, mortality and related costs incurred are on the rise, and there is a need for novel interventions; neuromodulation is one such area of research. In this article, we review various studies and existing evidence on BAT and VNS; and analyze in contrast to one another.

## Review

Sympathovagal imbalance in HF

The crux of neuromodulation of the heart is based upon the fact that the heart is an organ that is intricately regulated by the autonomic nervous system (ANS). The parasympathetic and sympathetic nervous systems make up the autonomic nervous system (ANS). Although these two physiologically opposing systems cooperate in a complementary way, they do so to be able to give the cardiac system the capacity to react fast to both intrinsic as well as extrinsic stimuli [8]. A decrease in the parasympathetic tone is noted in heart failure with decreased ejection fraction; however, there seems to be a growing corroboration that excitatory reflexes can also contribute to the autonomic disparity that exists in heart failure, despite the fact that these problems in autonomic regulation used to be previously accredited to a lack of the prohibitory input from arterial or cardiopulmonary baroreceptor reflexes [9-11].

The associations between the cardiac autonomic nervous system (ANS) central and peripheral components are the first mechanism through which neural regulation of heart function is exerted [12]. Secondly, the degree of the cardiac neuro-axis and the features of the primary heart disease can both influence how the mentioned interactions are reinforced or decreased [13-15]. Afferent inputs from anomalous sources are crucial for this type of neuronal remodeling [16-19]. Last but not least, because neuromodulation affects and the axons of passage, related neural networks, including the heart, the results of neuromodulation are dependent on the stimulation settings, the area of the cardiac neuro-axis to which treatment is given, and the cardio-neural pathological foundation against which the treatment is done [13].

Under usual circumstances, the primary inhibitors of sympathetic activity are the signals from non-baroreflex peripheral chemoreceptors and muscle "metaboreceptors," whereas the essential excitatory inputs to sympathetic outflow are "high pressure" carotid sinus and aortic arch baroreceptors and "low pressure" cardiopulmonary mechanoreceptors [4]. Additionally receptive to arterial baroreceptor afferent inhibitory input is the parasympathetic limb of the baroreceptor heart rate reflex. Thus, healthy individuals have minimal sympathetic discharge and substantial heart rate fluctuation while at rest. While peripheral baroreflex responses in HF patients are blunted as the condition progresses [4]. The central nervous system's sympathetic outflow is suppressed as a result of the dampening of the peripheral artery and cardiopulmonary baroreceptors, which also induces a rise in the efferent sympathetic stimulation and a decrease in the efferent parasympathetic tone [20]. As a result, HF patients have decreased heart rate variability and elevated peripheral vascular resistance [9].

Nerve stimulation therapy (VNS)

**Figure 1:**
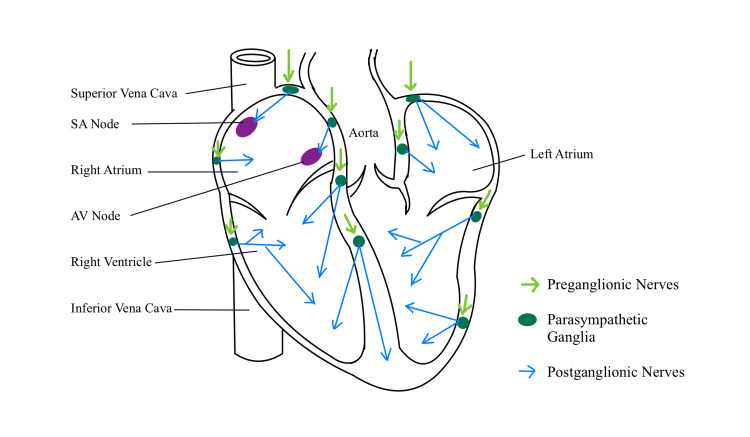
Vagus nerve action and sites of nerve receptor. SVC: superior vena cava, SAN: sinoatrial node, RA: right atrium, AVN: atrioventricular node, IVC: inferior vena cava, RV: right ventricle, PV: pulmonary vein, LA: left atrium, LV: left ventricle, VF: ventricular fibrillation. Image Credit: Manali Agrawal.

In order to understand the workings of VNS therapy in cardiac diseases, it becomes vitally important to understand the mechanism of vagal nerve stimulation corresponding to its innervation in cardiac muscle as shown in Figure 1 [21].

Vagal preganglionic nerves (left and right) are shown in light green. Shown in dark green are the synapses on a number of parasympathetic ganglia, which are located on the epicardium and in the atrial and ventricular septum. Postganglionic axons projected by parasympathetic ganglionic neurons are shown in blue. These innervations demonstrate the anatomical distribution of the axons, thereby shedding light on the physiological actions of vagal nerve stimulation. The location of innervation corresponding to the action produced by nerve stimulation is as follows: (1) sinoatrial node: lowering of heart rate and (2) atrioventricular node: reduction in contractility of ventricles, increase in ventricular fibrillation threshold [21].

This elaborate functioning of the vagal nerve makes it advantageous to use it in the form of therapy in the case of certain cardiac diseases. Various experimental studies have pointed out different cardioprotective effects of vagal nerve stimulation as well. In relation to heart failure, VNS is known to decrease the total circulatory cytokine level, which are inflammatory markers found in heart failure [22]. VNS also has potent anti-fibrillatory effects, which reduce the incidence of ventricular fibrillation in cases of acute myocardial ischemia by a great deal [23]. Apart from this, VNS therapy is proven to be anti-apoptotic and augments the expression of nitric oxide, which is a potent vasodilator. VNS inhibits and curtails norepinephrine release along with stellate ganglion activity, improves baroreceptor activity, suppresses macrophage activation, and production of other inflammatory markers. These diverse functions have been proven to protect against various cardiac conditions such as heart failure, myocardial infarction, and others [24-27].

VNS is generated with the help of a device named CardioFit 5000 that is made of an implantable neurostimulator and two leads, one of which is connected to an electrode that senses the QRS complex in the right ventricle, and the other one stimulates the right cervical vagal nerve. The vagal electrode is designed in a way that stimulates efferent fibers. This system provides a periodic pulse VNS at 1-3 Hz in a closed-loop manner [28,29].

However, recent clinical studies and trials have pointed towards the disadvantages of VNS therapy and how it is imperative to establish newer, more effective and alternative choices of therapy. The mixed results related to three major clinical trials regarding VNS therapy in heart failure with reduced ejection fraction (HFrEF) are needed to be discussed further. These studies are aimed at differentiating the VNS efficacy and functions in three different domains and are named as per the entities tested, such as in autonomic regulation therapy to enhance myocardial function and reduce progression of heart failure (ANTHEM-HF) function in autonomic regulation therapy via the left or right cervical vagus nerve stimulation in patients with chronic heart failure is studied, same is the case with neural cardiac therapy for heart failure (NECTAR-HF) and increase of vagal tone in heart failure (INOVATE-HF) [28]. The methodologies used in these trials were very different in terms of neurological targets, delivery of VNS, and technological platforms. The ANTHEM-HF was a randomized, multicenter uncontrolled study consisting of 60 patients. A delivery of 10 Hz VNS was used, and factors like mean heart rate, heart rate variability, and others were analyzed. It was initiated with the help of the open-loop cyberonics system in India and patients of NYHA classes II and III with left ventricular ejection fraction (LVEF) less than 40%. The outcome was fairly favorable as left ventricular ejection fraction considerably improved in the whole population [6,29]. The NECTAR-HF was also a multicenter, randomized sham-controlled study consisting of 96 people and 20 Hz VNS delivery was applied. In INOVATE-HF, 707 people were involved in a multinational, randomized controlled study that used high-amplitude and low-frequency VNS delivery. Despite the great potential and settings of these two studies, no significant improvements were observed in heart rate dynamics, and substantial evidence was generated in favor of VNS therapy by these randomized clinical trials. Rather, the results were neutral and not positive unlike in the ANTHEM trial. All the results were either neutral or disparate, hence not significant enough to prove the mantle of VNS as a recurring therapy in heart failure patients [4,13,28-32].

Apart from this, various studies have also shown the side effects of VNS, which should be considered. The most detrimental side effect of VNS is bradycardia, which eventually leads to a fatal condition called asystole. In asystole, the entire electrical activity of the heart gets stopped [33]. Patients might experience syncope, light-headedness, unconsciousness, or dyspnea after the implantation of the VNS device. This was established in a 2014 case report where all these symptoms resolved at the deactivation of VNS [34]. Infection is another complication associated with the implantation of VNS devices. Postoperative infections are noticed in 3% to 6% of patients. Some patients might also suffer from pain and hoarseness, which is a result of the left cord paralysis seen with VNS implantation.

Facial weakness was also attributed to VNS devices. These symptoms get resolved with the removal of the device. These risks tend to negate the argument of using VNS as the absolute management therapy in HFrEF patients [28].

Baroreceptor activation therapy

Device-based autonomic modulation, which is the basis of baroreceptor activation therapy (BAT), results in a decrease in sympathetic outflow and a rise in parasympathetic activity, which restores the autonomic nerve system's balance [35]. The delivery of BAT involves an implantable device that modifies the body's innate cardiovascular equilibrium by communicating with the brain through an electrode placed to the exterior of the carotid artery, which consecutively triggers the action of stabilizing sympathetic and parasympathetic operations to recover equilibrium [36]. The device is mainly designed for class III or class II NYHA patients with the latest presentation of class III who have an LVEF below 35% [36]. By stimulating the carotid baroreceptor with BAT, the sympathetic outflow is decreased centrally while parasympathetic activity is raised, increasing arterial and venous compliancy and decreasing peripheral resistance. BAT has also been demonstrated to be secure and efficient for decreasing high blood pressure in people with resistant hypertension (HTN) [37].

A device that resembles a pacemaker (Barostim Neo System, CVRx, Inc., Minneapolis, Minnesota, USA) delivers BAT. The lead consists of a 40-cm lead body that ends in a 7 mm-diameter circular backer. A 2-mm platinum-iridium disc electrode with an iridium oxide coating is positioned in the center of the backer. The pulse generator is embedded subcutaneously in an infraclavicular chest wall area, much like a pacemaker [35]. Transverse cervical incisions are made over the carotid bifurcation to expose the carotid sinus in preparation for electrode implantation.

After that, the electrode is temporarily positioned in several sites in the sinus region, and electrical stimulation is used to determine which area has the most sensitivity to BAT [33].

BAT therapy aims to restore the neurohormonal balance in patients suffering from heart failure with decreased ejection fraction by activating the carotid bifurcation [10,38]. The consolidated efferent reaction is produced and sent to the heart and blood vessels, reducing the level of excessive sympathetic activity, once the reaction obtained from the arterial vascular bed arrives at the nerve centers in the medulla oblongata [35].

A randomized, multicenter, prospective, controlled clinical trial called baroreflex activation therapy for heart failure (BEAT-HF) was conducted to evaluate the safety and efficacy of BAT as a therapeutic approach. In the BAT group, patients were randomized at random to receive both BAT and guideline-based medical therapy, whereas the control group received just guideline-based medical management. The results were encouraging and revealed significant improvements in NT-proBNP, the six-minute hall walk test, and quality of life [39].

A difficult priority is delineating the ideal patient population for BAT and, generally, for neuromodulation [40]. Despite receiving the best evidence-based, guideline-directed therapy, heart failure development is defined by deteriorating and repeated hospitalizations, along with increasing worsening and higher mortality [41]. A crucial clinical sign that should prompt doctors to investigate innovative treatments outside of disease management and pharmacological and device therapy in accordance with guidelines is recurrent heart failure hospitalizations [42]. It might be challenging to determine which subgroup of patients with numerous comorbidities will experience a clear benefit from BAT. The severity of symptoms in these patients may be more a function of the comorbidities present than of the heart failure itself. There may be a theoretical basis for measuring baroreflex dysfunction in certain patient groups using a clinical test like the phenylephrine test [35]. End-stage or unstable heart failure patients might be in an unrepairable disease state that prevents BAT from having a positive therapeutic effect. Patients who have acute pulmonary edema, permanent NYHA class IV heart failure symptoms, or who require IV inotropic medication are not the best candidates for BAT [35]. Patients with autonomic neuropathy or baroreflex dysfunction may have a low chance of benefiting from BAT. Implanting devices could mean that patients with autonomic neuropathy or baroreflex dysfunction may have a low chance of benefiting from BAT. Patients who have had prior surgery, radiation, or endovascular stent insertion in the carotid sinus region may have complicated implant procedures, which could make it more difficult to implant the carotid sinus lead. When major coexisting morbidities, such as severe asthma, chronic lung disease, or active cancer, are the primary cause of symptoms, the likelihood of benefiting may be low [35]. BAT should be used after reaching euvolemic status in order to effectively activate the vagus nerve because, while central venous pressure is still elevated, renal intraparenchymal pressure is increased due to venous backpressure, which hydrostatically increases pressure in the glomerulus and, in turn, increases sympathetic tone at a position too far along the signaling pathway for BAT to actually have an impact [43].

In 11 patients with severe heart failure who were at heightened risk of hospital readmission while receiving guideline-directed medication, a complete proof of concept research was carried out using BAT alone [44]. Due to the combination of pathophysiological and clinical information it offered, this study was significant for the development of BAT use [44]. Muscle sympathetic nerve activity (MSNA) was assessed in these 11 heart failure patients both at rest and sporadically following BAT stimulation. At three months, MSNA dramatically decreased and continued to decline at six months [44]. This finding continued after 21 months of follow-up and was accompanied by an extremely notable decrease in the number of hospital days compared to the year prior to BAT [45]. In a recent randomized controlled trial, the effectiveness of BAT was assessed in 140 patients with New York Heart Association (NYHA) class III heart failure and decreased ejection fraction who were either receiving only guideline-directed medical therapy or guideline-directed medical therapy and BAT. This clinical investigation's goal was to assess the CVRx Barostim Neo System's security and effectiveness in treating patients with heart failure using surrogate endpoints. BAT was linked to a tendency toward fewer days spent in hospitals for heart failure that was getting worse and considerably decreased N-terminal pro-brain natriuretic peptide [46,47]. Given that baroreceptor activation therapy controls autonomic stability and, more crucially, has a security profile resembling that of a pacemaker, it appears to be a promising therapeutic alternative [48]. We have tried to incorporate the comparison between various studies involved in vagal nerve stimulation in Table 1.

**Table 1:** Comparison of major studies involved in vagal nerve stimulation. NYHA: New York Heart Association, HF: heart failure, VNS: vagal nerve stimulation, BAT: baroreceptor activation therapy, FDA: Food and Drug Administration, ANTHEM-HF: autonomic regulation therapy to enhance myocardial function and reduce progression of heart failure, NECTAR-HF: neural cardiac therapy for heart failure, BEAT-HF: baroreflex activation therapy for heart failure, INOVATE-HF: increase of vagal tone in heart failure.

Parameters	ANTHEM-HF [30]	NECTAR-HF [49]	INNOVATE-HF [50]	BEAT-HF [39]
Number of patients	60	96	707	264
Average age	51	59	61	62
Design	Multi-center, open-label, phase II, randomized clinical trial	Phase II, multi-center Sham-controlled study	Phase III international, multi-center, randomized trial	Randomized trial
Primary endpoint	Ratio of deaths to the hospitalization rates in HF	Left ventricular end-systolic diameter (LVESD)	Ratio of deaths to the hospitalization rates in HF	Changes in a six-minute stress test over six months, proBNP levels
LVEF values in patients before starting the treatment regimen	≤40%	≤35%	≤40%	≤35%
Change in LVEF values in patients six months after treatment (in %)	+4.5	+0.9	0	N/A
Six-minute walk test (in meters) in trial patients	150-425 m	-	125-400 m	150-450 m
Change in six-minute walk test six months after starting treatment (in meters)	+56	N/A	+33	+60
Outcome	Improvements were seen in the hospitalization rates and death ratios and also in the secondary endpoint outcomes of NYHA class and six-minute walk distance.	Notable improvement in quality of life and NYHA class. Left ventricular systolic volume decreased in the crossover group (VNS off→on; 144 ± 37 to 139 ± 40, p>0.05) and LVEF (33.2 ± 4.9 to 33.3 ± 6.5, p>0.05)	Improvements were seen in the hospitalization rates and death ratios and also in the secondary endpoint outcomes of NYHA class and six-minute walk distance.	Considerable decrease of all components of primary efficacy endpoint. Based on it, BAT was approved by the FDA for use. 6 MHW distance increased (Δ = 60 m; 95% CI: 40 to 80 m; p<0.001), NT-proBNP decreased (Δ = −25%; 95% CI: −38% to −9%; p = 0.004)

## Conclusions

In view of the above contrasting review, it is quite evident that novice approaches have been effective in heart failure patients. Although VNS therapy has been proven advantageous by certain studies, its risks and side effects have also been well established by others. This makes the use of VNS therapy as the mainstay treatment extremely redundant. In addition to that, all the latest and relevant research studies have found BAT therapy to be a better option for the treatment of individuals associated with heart failure with reduced ejection fraction. However, this does not seem to be the endpoint as far as research in relation to the most optimal management therapy for heart failure is considered. Ideal population selection and rehospitalizations continue to remain the unresolved areas for BAT therapy. This hints towards underlying unexplored and ambiguous areas of BAT therapy that still need to be addressed. Further analytical studies and clinical trials are the answer for making the most out of the untapped potential of BAT therapy as the mainstay of heart failure therapy.

## References

[1] Dusi V, Angelini F, Zile MR, De Ferrari GM (2022). Neuromodulation devices for heart failure. Eur Heart J Suppl.

[2] Duncker D, Bauersachs J (2022). Current and future use of neuromodulation in heart failure. Eur Heart J Suppl.

[3] Chatterjee NA, Singh JP (2015). Novel interventional therapies to modulate the autonomic tone in heart failure. JACC Heart Fail.

[4] Byku M, Mann DL (2016). Neuromodulation of the failing heart: lost in translation?. JACC Basic Transl Sci.

[5] Guckel D, Eitz T, El Hamriti M (2023). Baroreflex activation therapy in advanced heart failure therapy: insights from a real-world scenario. ESC Heart Fail.

[6] Nearing BD, Anand IS, Libbus I, Dicarlo LA, Kenknight BH, Verrier RL (2021). Vagus nerve stimulation provides multiyear improvements in autonomic function and cardiac electrical stability in the ANTHEM-HF study. J Card Fail.

[7] Konstam MA, Mann DL, Udelson JJ (2022). Advances in our clinical understanding of autonomic regulation therapy using vagal nerve stimulation in patients living with heart failure. Front Physiol.

[8] Armour JA (2004). Cardiac neuronal hierarchy in health and disease. Am J Physiol Regul Integr Comp Physiol.

[9] Floras JS (2009). Sympathetic nervous system activation in human heart failure: clinical implications of an updated model. J Am Coll Cardiol.

[10] Hull SS Jr, Evans AR, Vanoli E (1990). Heart rate variability before and after myocardial infarction in conscious dogs at high and low risk of sudden death. J Am Coll Cardiol.

[11] La Rovere MT, Pinna GD, Hohnloser SH (2001). Autonomic tone and reflexes after myocardial infarcton. Baroreflex sensitivity and heart rate variability in the identification of patients at risk for life-threatening arrhythmias: implications for clinical trials. Circulation.

[12] Hanna P, Shivkumar K, Ardell JL (2018). Calming the nervous heart: autonomic therapies in heart failure. Card Fail Rev.

[13] Rajendran PS, Nakamura K, Ajijola OA, Vaseghi M, Armour JA, Ardell JL, Shivkumar K (2016). Myocardial infarction induces structural and functional remodelling of the intrinsic cardiac nervous system. J Physiol.

[14] Ajijola OA, Hoover DB, Simerly TM (2017). Inflammation, oxidative stress, and glial cell activation characterize stellate ganglia from humans with electrical storm. JCI Insight.

[15] Liu K, Li D, Hao G (2018). Phosphodiesterase 2A as a therapeutic target to restore cardiac neurotransmission during sympathetic hyperactivity. JCI Insight.

[16] Shivkumar K, Ajijola OA, Anand I (2016). Clinical neurocardiology defining the value of neuroscience-based cardiovascular therapeutics. J Physiol.

[17] Ardell JL, Andresen MC, Armour JA (2016). Translational neurocardiology: preclinical models and cardioneural integrative aspects. J Physiol.

[18] Wang HJ, Wang W, Cornish KG, Rozanski GJ, Zucker IH (2014). Cardiac sympathetic afferent denervation attenuates cardiac remodeling and improves cardiovascular dysfunction in rats with heart failure. Hypertension.

[19] Zucker IH, Patel KP, Schultz HD (2012). Neurohumoral stimulation. Heart Fail Clin.

[20] Marin-Neto JA, Pintya AO, Gallo Júnior L (1991). Abnormal baroreflex control of heart rate in decompensated congestive heart failure and reversal after compensation. Am J Cardiol.

[21] Capilupi MJ, Kerath SM, Becker LB (2020). Vagus nerve stimulation and the cardiovascular system. Cold Spring Harb Perspect Med.

[22] Deswal A, Petersen NJ, Feldman AM, Young JB, White BG, Mann DL (2001). Cytokines and cytokine receptors in advanced heart failure: an analysis of the cytokine database from the Vesnarinone trial (VEST). Circulation.

[23] Vanoli E, De Ferrari GM, Stramba-Badiale M, Hull SS Jr, Foreman RD, Schwartz PJ (1991). Vagal stimulation and prevention of sudden death in conscious dogs with a healed myocardial infarction. Circ Res.

[24] De Ferrari GM, Crijns HJ, Borggrefe M (2011). Chronic vagus nerve stimulation: a new and promising therapeutic approach for chronic heart failure. Eur Heart J.

[25] Uemura K, Li M, Tsutsumi T (2007). Efferent vagal nerve stimulation induces tissue inhibitor of metalloproteinase-1 in myocardial ischemia-reperfusion injury in rabbit. Am J Physiol Heart Circ Physiol.

[26] Yancy CW, Jessup M, Bozkurt B (2017). 2017 ACC/AHA/HFSA focused update of the 2013 ACCF/AHA guideline for the management of heart failure: a report of the American College of Cardiology/American Heart Association Task Force on Clinical Practice Guidelines and the Heart Failure Society of America. J Am Coll Cardiol.

[27] Verrier RL, Libbus I, Nearing BD, KenKnight BH (2022). Multifactorial benefits of chronic vagus nerve stimulation on autonomic function and cardiac electrical stability in heart failure patients with reduced ejection fraction. Front Physiol.

[28] Anand IS, Konstam MA, Klein HU (2020). Comparison of symptomatic and functional responses to vagus nerve stimulation in ANTHEM-HF, INOVATE-HF, and NECTAR-HF. ESC Heart Fail.

[29] Dusi V, De Ferrari GM (2021). Vagal stimulation in heart failure. Herz.

[30] Premchand RK, Sharma K, Mittal S (2014). Autonomic regulation therapy via left or right cervical vagus nerve stimulation in patients with chronic heart failure: results of the ANTHEM-HF trial. J Card Fail.

[31] Anand I, Ardell J, Gregory D (2020). Effects of sympathetic blockade on responsiveness to VNS in patients with heart failure and reduced ejection fraction. J Am Coll Cardiol.

[32] Ardell JL, Nier H, Hammer M (2017). Defining the neural fulcrum for chronic vagus nerve stimulation: implications for integrated cardiac control. J Physiol.

[33] Ben-Menachem E (2001). Vagus nerve stimulation, side effects, and long-term safety. J Clin Neurophysiol.

[34] Pascual FT (2015). Vagus nerve stimulation and late-onset bradycardia and asystole: case report. Seizure.

[35] Gronda E, Francis D, Zannad F, Hamm C, Brugada J, Vanoli E (2017). Baroreflex activation therapy: a new approach to the management of advanced heart failure with reduced ejection fraction. J Cardiovasc Med (Hagerstown).

[36] Bisognano J, Schneider JE, Davies S (2021). Cost-impact analysis of baroreflex activation therapy in chronic heart failure patients in the United States. BMC Cardiovasc Disord.

[37] Abraham WT, Fisher WG, Smith AL (2002). Cardiac resynchronization in chronic heart failure. N Engl J Med.

[38] Mann JA, Abraham WT (2019). Cardiac contractility modulation and baroreflex activation therapy in heart failure patients. Curr Heart Fail Rep.

[39] Zile MR, Lindenfeld J, Weaver FA, Zannad F, Galle E, Rogers T, Abraham WT (2020). Baroreflex activation therapy in patients with heart failure with reduced ejection fraction. J Am Coll Cardiol.

[40] Mortara A, Vanoli E (2015). Baroreceptor activation therapy: the importance of targeting the right patient: who needs to be treated?. Eur J Heart Fail.

[41] Jain P, Massie BM, Gattis WA, Klein L, Gheorghiade M (2003). Current medical treatment for the exacerbation of chronic heart failure resulting in hospitalization. Am Heart J.

[42] Bello NA, Claggett B, Desai AS (2014). Influence of previous heart failure hospitalization on cardiovascular events in patients with reduced and preserved ejection fraction. Circ Heart Fail.

[43] Kostreva DR, Seagard JL, Castaner A, Kampine JP (1981). Reflex effects of renal afferents on the heart and kidney. Am J Physiol.

[44] Gronda E, Seravalle G, Brambilla G (2014). Chronic baroreflex activation effects on sympathetic nerve traffic, baroreflex function, and cardiac haemodynamics in heart failure: a proof-of-concept study. Eur J Heart Fail.

[45] Gronda E, Seravalle G, Trevano FQ (2015). Long-term chronic baroreflex activation: persistent efficacy in patients with heart failure and reduced ejection fraction. J Hypertens.

[46] Abraham WT, Zile MR, Weaver FA (2015). Baroreflex activation therapy for the treatment of heart failure with a reduced ejection fraction. JACC Heart Fail.

[47] Durukan AB, Gurbuz HA (2018). Carotid baroreceptor activation therapy for resistant hypertension and heart failure: a report of two cases. Pol J Thorac Cardiovasc Surg.

[48] Premchand RK, Sharma K, Mittal S (2019). Background pharmacological therapy in the ANTHEM-HF: comparison to contemporary trials of novel heart failure therapies. ESC Heart Fail.

[49] De Ferrari GM, Stolen C, Tuinenburg AE (2017). Long-term vagal stimulation for heart failure: eighteen month results from the NEural Cardiac TherApy foR Heart Failure (NECTAR-HF) trial. Int J Cardiol.

[50] Gold MR, Van Veldhuisen DJ, Hauptman PJ (2016). Vagus nerve stimulation for the treatment of heart failure: the INOVATE-HF trial. J Am Coll Cardiol.

